# Unraveling the Composition of the Root-Associated Bacterial Microbiota of *Phragmites australis* and *Typha latifolia*

**DOI:** 10.3389/fmicb.2018.01650

**Published:** 2018-08-02

**Authors:** Laura Pietrangelo, Antonio Bucci, Lucia Maiuro, Davide Bulgarelli, Gino Naclerio

**Affiliations:** ^1^Department of Biosciences and Territory, University of Molise, Campobasso, Italy; ^2^Plant Sciences, School of Life Sciences, University of Dundee, Dundee, United Kingdom; ^3^Department of Agricultural, Environmental and Food Sciences, University of Molise, Campobasso, Italy

**Keywords:** rhizoplane, bacteria, microbiota, biofilm, *Phragmites*, *Typha*, phytodepuration, wetlands

## Abstract

*Phragmites australis* and *Typha latifolia* are two macrophytes commonly present in natural and artificial wetlands. Roots of these plants engage in interactions with a broad range of microorganisms, collectively referred to as the microbiota. The microbiota contributes to the natural process of phytodepuration, whereby pollutants are removed from contaminated water bodies through plants. The outermost layer of the root corpus, the rhizoplane, is a hot-spot for these interactions where microorganisms establish specialized aggregates designated biofilm. Earlier studies suggest that biofilm-forming members of the microbiota play a crucial role in the process of phytodepuration. However, the composition and recruitment cue of the *Phragmites*, and *Typha* microbiota remain poorly understood. We therefore decided to investigate the composition and functional capacities of the bacterial microbiota thriving at the *P. australis* and *T. latifolia* root–soil interface. By using 16S rRNA gene Illumina MiSeq sequencing approach we demonstrated that, despite a different composition of the initial basin inoculum, the microbiota associated with the rhizosphere and rhizoplane of *P. australis* and *T. latifolia* tends to converge toward a common taxonomic composition dominated by members of the phyla Actinobacteria, Firmicutes, Proteobacteria, and Planctomycetes. This indicates the existence of a selecting process acting at the root–soil interface of these aquatic plants reminiscent of the one observed for land plants. The magnitude of this selection process is maximum at the level of the rhizoplane, where we identified different bacteria enriched in and discriminating between rhizoplane and rhizosphere fractions in a species-dependent and -independent way. This led us to hypothesize that the structural diversification of the rhizoplane community underpins specific metabolic capabilities of the microbiota. We tested this hypothesis by complementing the sequencing survey with a biochemical approach and scanning electron microscopy demonstrating that rhizoplane-enriched bacteria have a bias for biofilm-forming members. Together, our data will be critical to facilitate the rational exploitation of plant–microbiota interactions for phytodepuration.

## Introduction

Wetlands can be defined as wet areas during a part or all the year at the interface zones between freshwater and soil (Srivastava et al., 2017). The complex wetlands ecosystem is based on the interaction between vegetation, microorganisms, animals, soil, and water. In recent years, the characterization of natural wetlands gained center stage owing to their contribution to the process of phytodepuration, whereby polluted sites are reclaimed to their natural status through the use of plants (Stout and Nüsslein, 2010; Faußer et al., 2012; Gupta et al., 2012; Sharma et al., 2013). Two plant species more represented in the natural wetlands and thus also more utilized in phytodepuration applications are *Phragmites* spp. and *Typha* spp. These plants can adapt to different abiotic conditions and, therefore, have a worldwide diffusion (Bellavance and Brisson, 2010; Li et al., 2013). In addition, these are perennial plants capable of performing the water cleaning process over consecutive years (Tsyusko et al., 2005; Mthembu et al., 2013; Srivastava et al., 2014; Bonanno and Cirelli, 2017; Eller et al., 2017) and finally, thanks to a rhizomatous propagation, can promptly colonize wetlands areas (Dhir, 2013; Juneau and Tarasoff, 2013). However, the molecular mechanisms underpinning the process of phytodepuration in wetlands are yet to be fully elucidated. Advances in sequencing technologies and computational analysis have revealed how plants host a myriad of microorganisms, collectively referred to as the microbiota, whose interactions at given plant sites define the plant microbiome (Schlaeppi and Bulgarelli, 2015). The characterization of the microbiota of land plants has been gaining momentum both in basic and translational science (Hacquard et al., 2015), yet elucidating the functional significance of the wetland plants microbiota is a research field in its infancy (Bowen et al., 2017; Cerri et al., 2017). For instance, roots of wetland plants offer a unique site of colonization with access to oxygen and organic resources for microorganisms otherwise thriving in water. Perhaps not surprisingly, certain microorganisms have evolved the capacity to exploit this opportunity by organizing themselves on the root surface in specific assemblages designated biofilm (Gagnon et al., 2007; Faußer et al., 2012; Srivastava et al., 2017). Likewise, the microbiota of wetland plants could contribute to the process of phytodepuration by enhancing plant uptake of mineral and organic compounds from the substrates, as suggested by the analogous role played by the microbiota of land plants (Alegria Terrazas et al., 2016). However, the composition of these assemblages and their potential contribution to phytodepuration remain largely unknown. A previous investigation suggests that Proteobacteria dominate the root–soil interface of *Phragmites australis* and *Typha latifolia* (Li et al., 2013). However, this investigation was conducted with low-resolution techniques and this makes it difficult to infer general principles. To gain novel insights into the wetland plants microbiota, here we present the characterization of the bacterial communities associated with the roots of *P. australis* and *T. latifolia*, which populate the natural area named “Le Mortine Oasis" (Campania, southern Italy). We tested the hypothesis that these plants shape the microbiota thriving at their root–soil interface. We adopted an Illumina MiSeq technology and we demonstrated that *Phragmites* and *Typha* assemble a rhizoplane microbiota whose taxonomic composition is significantly distinct from the surrounding water samples. Intriguingly, although differences between the microbial communities of the two tested species can be identified, *Phragmites* and *Typha* assemble a taxonomic congruent microbiota. Colonization patterns on the rhizoplane were confirmed using scanning electron microscopy (SEM). Next, we investigated the culturable portion of microorganisms in the rhizoplane specimens and we determined their biofilm formation potential. Overall, our data indicate that the wetland plants microbiota is not randomly assembled from the surrounding environment; rather plant-mediated mechanisms and the metabolic potential of the microbial communities thriving in water sculpt the microbiota at the *Phragmites* and *Typha* rhizoplane.

## Materials and Methods

### Samples Collection and Preparation

**FIGURE 1:**
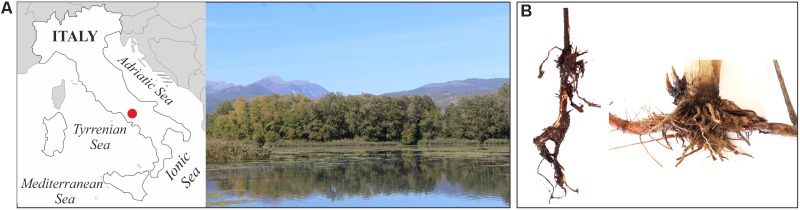
Sampling. Localization (**A**, left) and landscape (**A**, right) of the sampling site in the natural area of “Le Mortine Oasis” (41°28′11.4′′N 14°05′26.6′′E). Examples of *P. australis* (**B**, left) and *T. latifolia* (**B**, right) root systems sampled from the wetland.

Five root systems of *P. australis* and *T. latifolia* plants and four water samples (1 l) were collected from the wetland located in the naturalistic area of “Le Mortine Oasis” (Campania, southern Italy) (**Figure 1**). The samples were immediately transported to the laboratory in a portable cooler at approximately 4°C. Aliquots of the soil tightly adhering to *P. australis* and *T. latifolia* roots were collected in sterile Petri dishes and used to investigate the rhizosphere microbial communities. Likewise, water samples within 2 m radius from the plant’s sampling point were collected. Four root segments (approximately 2 cm in length and 0.3–0.5 cm in thickness) were obtained from each plant system. Subsequently, the root segments were washed by shaking three times in 10 ml of tap water, twice in 10 ml of distilled water, and once in 20 ml of sterile 0.85% NaCl (Kirzhner et al., 2009; Li et al., 2013).

### Biomolecular Analyses

Water samples (1 l) were filtered through sterile mixed esters of cellulose membranes (S-Pak^TM^ Membrane Filters, 47 mm diameter, 0.22 μm pore size, Millipore Corporation, Billerica, United States) and the DNA was extracted from the filters using the PowerWater^®^ DNA Isolation Kit (MO BIO Laboratories, Inc., Carlsbad, United States) following manufacturer’s recommendations. The DNA samples generated from the wetland water (W1, W2, W3, and W4) were stored at -20°C until further use. Approximately 0.5 g of each rhizosphere sample was subjected to the DNA extraction using the PowerSoil^®^ DNA Isolation Kit (MO BIO Laboratories, Inc., Carlsbad, United States) as per the manufacturer’s recommendations. DNA extracted from *P. australis* (*P*r1, *P*r2, *P*r3, and *P*r4) and *T. latifolia* (*T*r1, *T*r2, *T*r3, and *T*r4) rhizosphere was stored at -20°C until further use. Furthermore, three of the washed root segments were subjected to a double step ultrasound treatment using the Vibra-Cell^TM^ ultrasonic processor VCX 130 (Sonics & Materials, Inc., Newtown, United States) set at the constant frequency of 20 kHz and at the amplitude of 30%, with a 6 mm probe. Firstly, the root specimens were sonicated in 10 ml of sterile sonication buffer (0.85% NaCl and 0.1% Tween 80) for 2 min and 30 s in 15 ml Falcon tube, then root specimens were transferred into 10 ml new sterile sonication buffer and subjected to a second ultrasound treatment in the same conditions for 5 min. This procedure ensured through the first sonication step the removal of cells not firmly attached onto the root surface and the detachment and subsequent collection of the rhizoplane cells during the second step. After the ultrasound treatments, the roots were recovered and fixed using 3% glutaraldehyde in a 0.1 M phosphate buffer at pH 7.2 for 24 h. Therefore, samples were washed three times using the same buffer and dehydrated through an ethyl alcohol series (30, 50, 70, 95, and 100%, for 5 min at each step). After dehydration, they were dried using an Emitech K850 Critical Point Dryer (Quorum Technologies Ltd., United Kingdom), mounted on aluminum stubs and coated with gold using an Emitech K550 sputter coater (Quorum Technologies Ltd., United Kingdom). Finally, the prepared samples were observed using a ZEISS DSM-940A Scanning Electron Microscope (Carl Zeiss, Jena, Germany) at 10 kV and 30× and 2000× magnification images were acquired. Instead, the suspension of rhizoplane cells was divided in two aliquots (5 ml) representing replicates of each sample (indicated as “a” and “b”). The aliquots were brought to a final volume of 100 ml with sterile Milli-Q water and filtered through sterile mixed esters of cellulose membranes (S-Pak^TM^ Membrane Filters, 47 mm diameter, 0.22 μm pore size, Millipore Corporation, Billerica, United States). DNA was finally extracted from the filters using the PowerWater^®^ DNA Isolation Kit (MO BIO Laboratories, Inc., Carlsbad, CA, United States) following the recommended protocol. More precisely, for each biofilm suspension two DNA samples were originated as replicates and they were indicated as “a” and “b,” respectively. A total of 10 DNA samples were obtained from the rhizoplane of *P. australis* (*P*1a and *P*1b; *P*2a and *P*2b; *P*3a and *P*3b; *P*4a and *P*4b; *P*5a and *P*5b) and *T. latifolia* (*T*1a and *T*1b; *T*2a and *T*2b; *T*3a and *T*3b; *T*4a and *T*4b; *T*5a and *T*5b). The DNA samples were stored at -20°C until further use. DNA samples were quantitated using the NanoDrop 1000 spectrophotometer (Thermo Fisher Scientific, Wilmington, United States) and they were subjected to the amplification of the hypervariable V4 region of the 16S rRNA gene through a nested-PCR approach to generate amplicon libraries. For each plant species, we therefore processed four biological replicates of rhizosphere specimens, five biological replicates of rhizoplane specimens, and two biological replicates of water samples. The PCRs were performed using Kapa HiFi HotStart PCR kit (Kapa Biosystems, Wilmington, United States) in a G-Storm GS1 Thermal Cycler (Gene Technologies, Somerton, United Kingdom). For the first amplification step, the PCR mix contained 50 ng of DNA, 4 μl of 5× Kapa HiFi Buffer, 10 ng bovine serum albumin (Roche, Mannheim, Germany), 0.6 μl of a 10 mM Kapa dNTPs solution, 0.6 μl of 10 μM solutions of the 27F (5′-AGAGTTTGATCMTGGCTCAG-3′) and 1392R (5′-ACGGGCGGTGTGTRC-3′) PCR primers, 0.25 μl of Kapa HiFi polymerase and sterile Milli-Q water up to the final volume of 20 μl. The reaction was performed with an initial denaturation at 94°C for 3 min, then 20 cycles of denaturation at 98°C for 30 s, annealing at 55°C for 30 s, elongation at 72°C for 1 min and 30 s, and a final elongation step at 72°C for 10 min. The second amplification step was conducted using 2 μl of the first amplification product as template, 4 μl of 5× Kapa HiFi Buffer, 10 ng bovine serum albumin (Roche, Mannheim, Germany), 0.6 μl of a 10 mM Kapa dNTPs solution, 0.6 μl of 10 μM solutions of the 515F (5′-GTGCCAGCMGCCGCGGTAA-3′) and 806R (5′-GGACTACHVGGGTWTCTAAT-3′) PCR primers, 0.25 μl of Kapa HiFi polymerase and sterile Milli-Q water up to the final volume of 20 μl. To generate the amplicon libraries, both primers used in this PCR step presented flow cell adapter sequences at their 5′ termini and the primers 806R also 12-mer unique “barcode” sequences to provide the simultaneously sequencing of several samples (Caporaso et al., 2012). This PCR was performed using the following conditions: initial denaturation at 94°C for 3 min, denaturation at 98°C for 30 s, annealing at 50°C for 30 s, elongation at 72°C for 1 min for a total of 25 cycles and a final elongation step at 72°C for 10 min. Reaction negative controls (rNTCs) were generated in all the individual PCRs and for all the barcodes used in the second amplification. Furthermore, four no-template samples were amplified through both nested-PCR steps and thus they were tagged by their own barcodes in the second amplification step to be used as sequencing negative controls (sNTCs). Five microliters of amplified samples and controls were checked on 1.5% agarose gel. The samples which showed the expected size amplicon and whose rNTCs presented no detectable amplicon were used for the amplicons library construction. The four sNTCs were also used to generate the amplicons library. The amplicons and the sequencing negative controls (sNTCs) were purified using Agencourt AMPure XP kit (Beckman Coulter, Brea, United States) with a ratio of 0.7 μl AMPure XP beads per 1 μl of sample and then 3 μl of each sample were quantified using Picogreen (Thermo Fisher Scientific, United Kingdom) according to the manufacturer’s recommendations. After that, individual barcode samples were pooled at equimolar ratios to generate the amplicon libraries. All library QC and processing was carried out by the Genome Technology group at James Hutton Institute (Invergowrie, United Kingdom).

### OTU Table Generation

Sequencing reads produced by the MiSeq machine were processed in QIIME, version 1.9.0 (Caporaso et al., 2010) using default parameters. Specific options adopted for the individual commands are indicated below. Briefly, ***f***orward and ***r***everse read FASTQ files were decompressed and merged into overlapping paired-end reads (minimum overlap imposed 5 bp) using the command join_paired_ends.py. We used the command split_libraries_fastq.py to assign overlapping paired-end reads to individual samples. Only overlapping reads with a minimum PHRED score of 20 were retained for the analysis. These high-quality PE reads were used to define operational taxonomic units (OTUs) a 97% sequence identity. OTUs were identified using the ***“***closed reference***”*** approach against the chimera-checked Greengenes database (DeSantis et al., 2006), version 13_5. Individual OTUs were defined using the SortMeRNA OTU-picking algorithm (Kopylova et al., 2012). Singleton OTUs, i.e., OTUs with a single sequencing count in the whole dataset, were removed using the command filter_otus_from_otu_tables.py. Rarefied OTU tables (see below) were used to generate phylum taxonomy charts using the function summari***z***e_taxa_through_plots.py.

### Data Analysis

The obtained OTU table was statistically analyzed in R (version 3.4.2) using the R Phyloseq package (McMurdie and Holmes, 2013). The alpha- and beta-diversity calculations were performed for two samples sets in parallel, each one composed of all the rhizosphere and water samples plus respectively the first set of rhizoplane replicates (set1) or the second set of replicates (set2). Therefore, two independent OTUs tables were obtained. Firstly low abundance OTUs were filtered from the datasets, referring to those OTUs observed for less than 25 reads in at least the 20% of samples; then the residual reads were rarefied at the sequencing depth of 66,000 sequencing reads per sample. After filtering, we obtained 1,906 unique OTUs for the samples set1 and 1,901 for the samples set2. For the alpha-diversity calculation, the richness within samples was evaluated through number of observed OTUs and Chao1 index whereas the evenness was estimated through Shannon index. Data were visualized using the function ggplot from the package ggplot2. For each dataset, the normality of rhizosphere and rhizoplane data distribution was evaluated using the Shapiro–Wilk test to evaluate the microbial diversity between the two more closely related microhabitats. We imposed the alpha level to infer whether the data tested were normally distributed establishing a *p*-value <0.01 for the richness parameters, i.e., observed OTUs and Chao1 index, and a *p*-value <0.05 for the evenness calculation through Shannon index. For datasets whose Shapiro–Wilk test generated a *p*-value lower than the established alpha levels, and consequently resulted not normally distributed, a non-parametric analysis of variance was performed through Wilcoxon test to evaluate the microhabitat effect on the microbial diversity. For the beta-diversity calculation firstly the OTU counts were transformed to relative abundance and then the distance between samples was calculated using both the Bray–Curtis index, which is sensitive to the OTU relative abundance only, and the weighted UniFrac index, sensitive to OTU relative abundance and also to phylogenetic assignment (Lozupone and Knight, 2005). Distance matrices were represented through principal coordinates analysis (PCoA). In order to evaluate the effect of microhabitat and plant species on community composition, distance matrices were subjected to an analysis using the adonis function of the package vegan and *p*-values were calculated for 5,000 permutations. Furthermore, a differential analysis of the count data was executed to identify individual bacteria differentially recruited between the rhizoplane and the rhizosphere of the two studied plants using negative binomial generalized linear models and the package DESeq2 (Love et al., 2014). For each samples set, the OTU count and sample information were collapsed to generate a DESeq object. We considered as rhizoplane-enriched OTUs of *P. australis* and *T. latifolia* only those resulted being enriched respect to the rhizosphere in both the samples sets. Then we compared the rhizoplane-enriched OTUs of the two plant species between each other and we enumerated and identified the conserved ones as the OTUs enriched in the rhizoplane of both plants. Therefore, to compare the proportion of enriched OTUs in the rhizoplane of each and both plants we generated a Venn diagram using the R package VennDiagram. The complete script used to perform the data analysis of the present study and to generate the related figures is available at https://github.com/BulgarelliD-Lab.

### Culture-Dependent Approach

For each studied plant species, a composite sample of five roots was generated pooling one washed root from each root system. The five root samples were subjected to the ultrasound treatment following the same procedure described above. After the ultrasound treatment, the roots were removed and the rhizoplane suspension was divided in two series of 4 ml, 400 μl, 40 μl, and 4 μl aliquots. Sterile Milli-Q water was added to the aliquots to reach the final volume of 100 ml. The samples were filtered through sterile mixed esters of cellulose membranes (S-Pak Membrane Filters, 47 mm diameter, 0.22 μm pore size, Millipore Corporation, Billerica, United States). The filters obtained were placed on 2% Nutrient (Difco-BD, Sparks, United States) and R2A (Lab M, Lancashire, United Kingdom) agar media and plates were incubated for 48 h at 25 and 37°C, respectively (Calheiros et al., 2009; Kirzhner et al., 2009). An aliquot of the samples (50 μl) was also directly spread without filtering on the surface of each medium and incubated in the same conditions. After incubation, the colonies grown on the membrane surface were discriminated on the basis of their morphological characteristics, color, and size. The selected colonies were picked and re-streaked onto 2% agarised TY medium (1.6% tryptone, 1% yeast extract, 0.5% NaCl) to obtain pure cultures. Then, the isolated colonies capable of growth at both 25 and 37°C were inoculated in TY broth at higher temperature in shaking condition (200 rpm) and culture aliquots were stored in 20% glycerol stocks at -80°C originating a collection of rhizoplane isolates for each plant species. The isolates were tested for their ability to form biofilm *in vitro* through a modified Stepanović biofilm formation assay (Stepanović et al., 2004). They were statically grown over-weekend in TY broth at room temperature and subsequently their optical density (O.D.) was measured using the spectrophotometer UV-1601 (SHIMADZU, Kyoto, Japan) at the wavelength of 600 nm. The cultures were diluted in triplicates to the O.D. of 0.2 in a final volume of 0.2 ml of TY broth in sterile 0.5 ml Eppendorf tubes. Also controls were generated in triplicates using 0.2 ml of TY broth only. The replicates of each isolate and also the controls were incubated statically at 37°C for 24, 48, and 72 h, respectively. At the end of each incubation time, the culture was removed and the tubes were washed three times with 300 μl of distilled water. The biofilm attached to the tube walls was fixed with 250 μl of methanol. After 15 min, the tubes were emptied and dried under the laminar flow hood. The dried tubes were stained with 250 μl of 2% Crystal violet solution from the Gram staining kit (Biolife Italiana srl, Milan, Italy) for 5 min. The excess of stain was removed firstly using a pipette and then rinsing out under flowing tap water. The tubes were dried in upside down position under the laminar flow hood. Subsequently, 250 μl of 33% (v/v) glacial acetic acid was added to redissolve the dye entered in the biofilm cells. The solution was transferred to a spectrophotometric 2 ml plastic cuvette and 33% (v/v) glacial acetic acid was added to reach the final volume of 1 ml. The O.D. at 570 nm was measured using the spectrophotometer UV-1601 (SHIMADZU, Kyoto, Japan) and compared to the O.D. measured for the controls. According to Stepanović et al.’s (2004) protocol and classification, the cut-off O.D. (O.D.c) was calculated as three standard deviations above the mean O.D. measured for the negative controls and the rhizoplane isolates were classified as: no biofilm producer when O.D. ≤ O.D.c, weak biofilm producer if O.D.c < O.D. < (2.O.D.c), moderate biofilm producer when (2.O.D.c) < O.D. < (4.O.D.c), and strong biofilm producer in case of (4.O.D.c) < O.D. Some of the isolates which resulted able to form biofilm *in vitro* test were identified. Their 16S rRNA gene was amplified and sequenced through Bact16S protocol at BMR genomics (University of Padova, Italy). The obtained sequences were matched with the Greengenes database obtaining the identification for each considered isolate.

## Results

### Composition of the Prokaryotic Communities Associated to *P. australis* and *T. latifolia*

**FIGURE 2:**
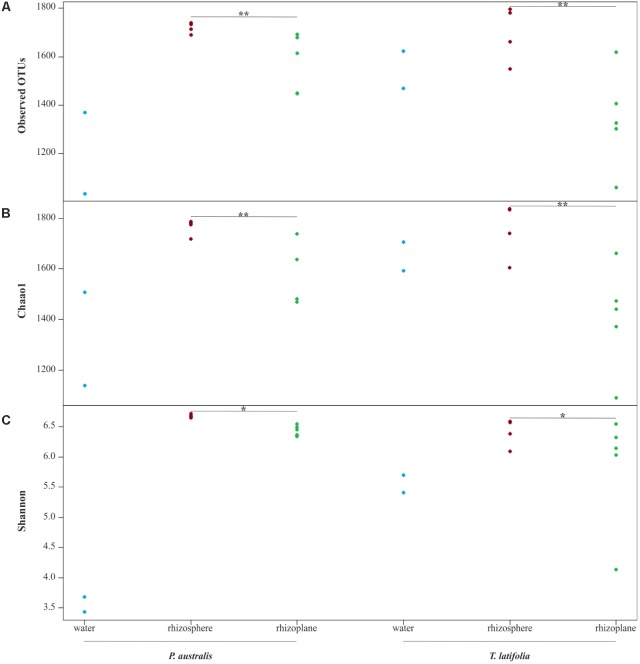
Alpha diversity calculation. OTUs richness of water, rhizosphere, and rhizoplane microbiotas of *Phragmites australis* and *Typha latifolia* indicated by number of observed OTUs **(A)** and by Chao1 index **(B)**. The OTUs evenness of the two plants microbiotas is shown by Shannon index **(C)**. Dots represent single samples. Asterisks denote statistically significant differences between rhizosphere and rhizoplane microhabitats (^∗∗^*p* < 0.01, ^∗^*p* < 0.05).

**FIGURE 3:**
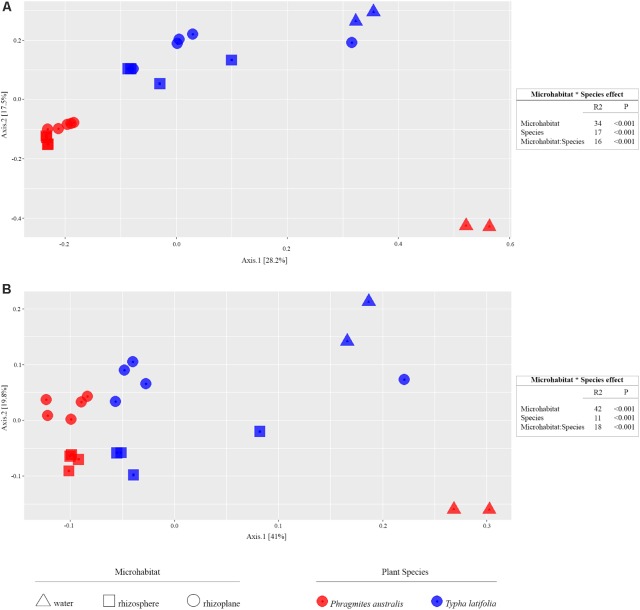
Beta-diversity calculation. On the left, the PCoA plots show the distance between samples calculated using Bray–Curtis index sensitive to the OTUs relative abundance (**A**, left) and weighted UniFrac index sensitive to both OTU relative abundances and taxonomic affiliation (**B**, left); the colors of shown symbols depict the plant species and their shapes indicate the considered microhabitats. On the right, the permutational analysis of variances for the indicated sources of variation calculated for the Bray–Curtis (**A**, right) and weighted UniFrac (**B**, right) indexes. The R2 value shows the proportional effect of the indicated factors in the samples distancing and the *p*-values were calculated for 5,000 permutations.

From the amplicon sequencing of 16S rRNA gene we obtained a total of 6,903,866 high quality reads. After *in silico* depletion of OTUs derived from plant mitochondrial and plastidial DNA the number of reads useful for downstream analysis decreased to 6,766,926 with a retaining percentage of 98%. In total we identified 15,436 OTUs at 97% sequence identity. To increase the efficiency of amplification of rhizoplane samples, we subjected all our specimens to a nested-PCR approach. However, this approach relies on a high number of cycles which might increase the proportion of PCR biases and spurious amplifications of environmental microbes (i.e., contaminations). To control for this source of variation, we decided to subject to sequencing also four no-template controls (sNTCs) and two sets of technical replicates of the rhizoplane specimens (hereafter, set1 and set2). We reasoned that a sequencing contamination would have been represented by a limited number of OTUs accounting for a large proportion of the sequences recovered from sNTCs samples. Consistent with our assumption, we identified 56 OTUs with a relative abundance equal or greater to 0.01% which accounted for the vast majority of the sNTCs sequencing profiles (>99%, Supplementary Datasheet 1). Next, we pruned these OTUs, and their assigned sequences, from the entire dataset. Remarkably, after removal of contaminant OTUs, we were able to retain more than 97% of the initial high quality reads assigned to water, rhizosphere, and rhizoplane samples (Supplementary Datasheet 1) indicating that the occurred contamination had a negligible impact on the profiling of the samples of interest. To further monitor the impact of the nested-PCR approach on the composition of the microbiota, we also inspected the composition of the profiles at high taxonomic rank (i.e., phylum level, Supplementary Figure 1 and Supplementary Datasheet 1). This analysis revealed that members of the phyla Acidobacteria, Actinobacteria, Bacteroidetes, and Proteobacteria dominated the microbiota of rhizosphere and rhizoplane samples, which is consistent with previous data obtained for the plant microbiota (Bulgarelli et al., 2013). Interestingly, we noticed a significant proportion of sequences assigned to members of the phylum Verrucomicrobia, in particular in water samples (∼8%, Supplementary Datasheet 1). This observation is congruent with the ubiquity (Zwart et al., 2003) and dominance (Chiang et al., 2018) of this phylum in lake habitats. Together these data suggest that, if any bias was introduced during the nested-PCR approach, this bias had a limited impact on the composition of the tested microbial communities. We therefore investigated alpha-diversity at OTU level considering the two sets of samples, each one composed of rhizosphere and water samples plus respectively the first set of rhizoplane replicates (set1) or the second set of replicates (set2). In detail, the OTUs richness was evaluated through the number of observed OTUs and Chao1 index whereas the OTUs evenness was evaluated through Shannon index. Since the results obtained for the two samples sets were consistent with each other, only those regarding the first set are treated below; the results obtained for the other samples set are shown in Supplementary Figure 2. The rhizoplane communities of *P. australis* and *T. latifolia* resulted being characterized by a mean value of observed OTUs of 1,581 and 1,337, respectively; more interestingly, both the OTUs richness (**Figures 2A,B**) and OTUs evenness (**Figure 2C**) of the rhizoplane communities resulted significantly different and lower than the rhizosphere communities (Wilcoxon test with *p* < 0.01 for observed OTUs and Chao1 index, with *p* < 0.05 for Shannon index). This result suggested clearly the specificity of the rhizoplane microbial community composition respect to the rhizosphere one for both the studied plants and, at the same time, it confirmed that the ultrasound treatment of root samples was effective to isolate selectively the rhizoplane microhabitat and microbial community. For both samples sets we generated Bray–Curtis distance matrix to evaluate the diversity on the base of OTUs relative abundance and then we also generated weighted UniFrac distance matrix which is sensitive at the same time to OTU relative abundance and phylogenetic classification. Also for the beta-diversity analysis, only the results regarding one sample set are illustrated below whereas the results obtained for the other sample set are shown by Supplementary Figure 3. The Bray–Curtis matrix showed a clear species effect manifested by segregation of samples along the axis accounting for the second source of variation. Conversely, on the axis explaining the largest variation in community composition a microhabitat effect was identified. Statistical analyses confirmed a further interaction between microhabitat and species in defying the composition of the microbiota (**Figure 3A**). Instead, the weighted UniFrac distance matrix showed a more pronounced microhabitat effect and a lower effect of the species compared to the Bray–Curtis distance matrix. In this case, the effect of the interaction between microhabitat and species was larger than the effect of species only (**Figure 3B**). These results suggest that, irrespective of the initial diversity of the starting inoculum, represented by the “water” microbiota, the *Phragmites*- and *Typha*-associated microbiotas tend to converge toward a similar composition. Yet, the distinct host-associated habitats, the rhizosphere and the rhizoplane, exert their selection pressure on microbes which is further fine-tuned by the individual plant species.

### Specific Taxa Are Enriched in the Rhizoplane Microbiota

**FIGURE 4:**
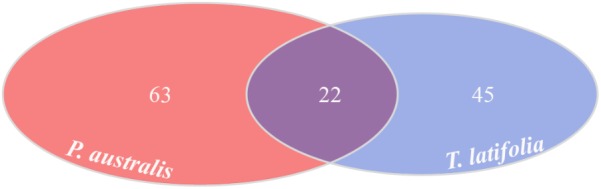
Rhizoplane-enriched OTUs. Number of OTUs enriched in the rhizoplanes of both studied plants (Wald test, FDR < 0.05)

**FIGURE 5:**
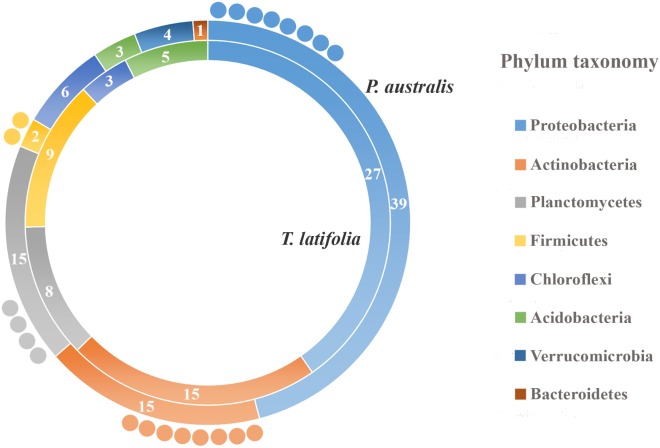
Phylum taxonomy of the OTUs enriched in the rhizoplane microbiota. Phyla significantly enriched in rhizoplane of *Phragmites australis* (outer ring) and *Typha latifolia* (inner ring) respect to the rhizosphere (Wald test, FDR < 0.05); the number of OTUs for the identified phyla is reported in the correspondent ring sectors. The external circles represent the number of OTUs enriched in the rhizoplane of both plant species.

Due to their intimate relationships with the host, the rhizoplane communities represented an attractive model to further characterize species-specific signatures of *Phragmites* and *Typha* on the microbiota. Firstly, for each plant species, we determined the number of rhizoplane-enriched OTUs as the pool of OTUs shared between the rhizoplane replicates (set1 and set2): a total of 85 enriched OTUs for *P. australis* rhizoplane and 67 for the *T. latifolia* one were identified (Wald test, FDR < 0.05 and log2FC > 0) as reported in Supplementary Table 1. Then, the identified enriched OTUs of *P. australis* and *T. latifolia* rhizoplane were compared each other: 22 OTUs resulted enriched both in *P. australis* and *T. latifolia* rhizoplane, whereas 63 OTUs were differentially enriched in *P. australis* rhizoplane, and 45 in the rhizoplane of *T. latifolia* (**Figure 4** and Supplementary Table 1). Subsequently, we evaluated the taxonomical composition at phylum level firstly of all the enriched OTUs in the *P. australis* and *T. latifolia* rhizoplane microbiotas then of the conserved OTUs only, referring to the OTUs enriched in the rhizoplane of both plant species. Among the enriched OTUs of *Phragmites* and *Typha* rhizoplane a total of eight and six phyla were identified, respectively (**Figure 5**). The rhizoplane microbiotas resulted dominated from the phyla of Proteobacteria, Planctomycetes, and Actinobacteria observed at similar proportions in both the plant species. On the contrary, the phyla of Acidobacteria and Firmicutes resulted more represented in the rhizoplane community of *T. latifolia* as well as the Chloroflexi phylum resulted more abundant in the rhizoplane microbiota of *P. australis*. Conversely the phyla of Verrucomicrobia and Bacteroidetes were differentially detected in the rhizoplane community of *P. australis* (**Figure 5**). Instead, regarding to the taxonomy of the OTUs enriched in both the rhizoplane microbiotas we verified that these conserved OTUs belonged mostly to the phyla of Proteobacteria, Planctomycetes, and Actinobacteria but also to the phyla of Firmicutes although they were represented at a lower proportion respect to the other detected phyla (**Figure 5**). Summarizing, all the results obtained from the taxonomical analysis of the enriched OTUs suggested that the phyla of Proteobacteria, Planctomycetes, Actinobacteria, and Firmicutes constitute a central core of the rhizoplane microbiota with which other phyla like the Acidobacteria, Bacteroidetes, Chloroflexi and Verrucomicrobia interact at different level and depending on the plant species.

### The Rhizoplane of *P. australis* and *T. latifolia* Is a Site for Microbial Colonization

**FIGURE 6:**
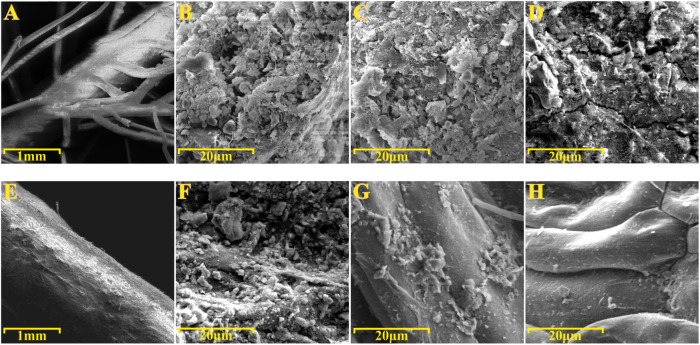
Scanning electron microscopy (SEM) of roots specimens before and after ultrasound treatments. SEM micrographs of **(A–D)** *Phragmites australis* and **(E–H)** *Typha latifolia* roots. **(A,B,E,F)** Root specimens before ultrasound treatments. **(C,G)** Rhizoplane of specimens subjected to the first ultrasound treatment. **(D,H)** Rhizoplane of specimens subjected to the second ultrasound treatment. Note the bacterial-like structure proliferating on the rhizoplane of both species.

To gain further insights into the spatial organization of the *P. australis* and *T. latifolia* rhizoplane microbiota, root specimens were subjected to the SEM analysis. SEM micrographs of washed roots revealed the presence of microbial-like structures and assemble colonizing the root surface of both studied plants (**Figures 6B,F**). Intriguingly, these microbial-like assemblages appeared more compacted and developed on the rhizoplane of *P. australis* (**Figure 6B**), since we repeatedly identified areas of *T. latifolia* rhizoplane uncovered by this microbial-like matrix (**Figure 6F**). SEM micrographs of the roots subjected to the sonication pre-treatment confirmed a progressive dislodgment of the microbial-like aggregates from the rhizoplane in both tested species (**Figures 6C,G**). This phenomenon was more evident on the specimens subjected to the second ultrasound treatment (**Figures 6D,H**). Collectively, these observations suggest that the rhizoplane of *P. australis* and *T. latifolia* is a site for microbial proliferation whose anchoring to the host cells is sufficiently robust to withstand water streams at the root surface, such as the ones occurring at the sites where these two plants develop. Likewise, these observations directly corroborate our assumption that ultrasound treatments would have enriched the sonication buffer for rhizoplane-colonizing microbes.

### Root-Isolated Bacteria Have Distinct Biofilm-Forming Capabilities

**FIGURE 7:**
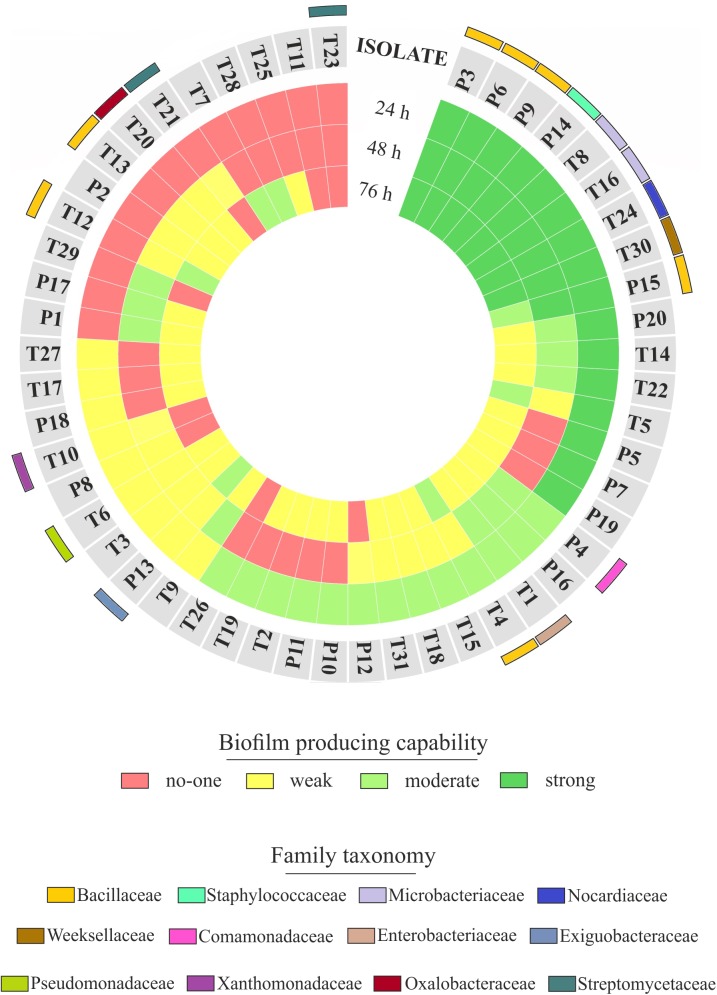
Rhizoplane bacterial isolates, their biofilm formation capability and taxonomical classification. The biofilm formation capability of the *Phragmites* (P) and *Typha* (T) rhizoplane isolates is represented by the three inner rings, which report the results of the biofilm formation assay for each isolate obtained during the indicated incubation times. The different colors exemplify the classification of those isolates as not, weak, moderate, or strong biofilm producers. The outer colored bars show the family affiliation of the identified isolates.

**FIGURE 8:**
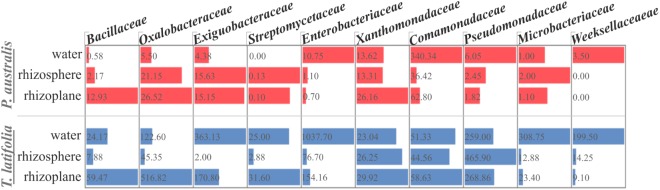
Proportional abundance of the microbial families whom the identified biofilm formers belong to, in water, rhizosphere, and rhizoplane microbiotas of *P. australis* and *T. latifolia*. Bars and inner numbers indicate the average number of reads observed for the microbial families in each microhabitat.

The observation of that tightly associated rhizoplane communities proliferate in the tested plants (see **Figure 6**) motivated us to investigate the biofilm-forming capabilities of *P. australis* and *T. latifolia* root-associated microbiota. We therefore, decided to isolate individual members of the rhizoplane-inhabiting microbiota on synthetic media. In total, we retrieved 20 morphologically distinct colony forming units (CFUs) from *P. australis* and 31 from *T. latifolia* and tested their biofilm-forming capabilities (**Figure 7**). Therefore, strong biofilm producers alongside with other 12 “control” isolates with different trends of biofilm formation were subjected to taxonomic identification using a 16S rRNA gene sequencing approach (Supplementary Table 2). Next, we identified an overlap between the higher rank taxonomies of the bacteria isolated from roots and bacteria identified in our sequencing survey as enriched in the rhizoplane microbiota of *P. australis* and *T. latifolia* (compare **Figure 5** with **Figure 7**). Interestingly, at family level only the Nocardiaceae and Staphylococcaceae families did not match with the families identified in the rhizoplane microbiotas but all the other identified families resulted effectively part of the rhizoplane microbiota of the two studied plants (**Figure 8**). These data suggest that the biofilm-forming bacteria isolated from rhizoplane preparations are phylogenetically related to members of the rhizoplane microbiota. This is particular evident for members of two bacterial families, namely Bacillaceae and Oxalobacteraceae, whose enrichment in the rhizoplane significantly discriminate this compartment from the microbiota surrounding *Phragmites* and *Typha* roots (**Figure 8**). To ultimately gauge the ecological significance of these observations it will be important to construct a bacterial collection encompassing more than the 51 isolates described in here, whose limited number might underestimate the real diversity of the culturable *Phragmites* and *Typha* microbiota.

## Discussion

The data presented here provide new insights into the composition of bacterial microbiota associated to *P. australis* and *T. latifolia*, two macrophytes commonly present in natural and artificial wetlands. Despite a consistent part of scientific literature is focused on the role of macrophytes and rhizosphere microorganisms in phytodepuration process, very little is known about the microbial communities which more tightly interact with root surface in the rhizoplane (Dhote and Dixit, 2009; Sharma et al., 2013; Shelef et al., 2013). In an attempt to unravel the composition and recruitment cues of the rhizoplane microbiota of the two plants we used a combined culture independent- and dependent-approach. Through a 16S rRNA gene Illumina MiSeq sequencing survey we characterized the rhizoplane communities of the two plants and compared them to the microbial communities of the surrounding water and rhizosphere. Alpha diversity (i.e., within sample diversity) calculation indicated that, regardless of the tested plant species, the rhizoplane bacterial microbiota of the two plants was significantly different from the one retrieved from rhizosphere specimens (**Figure 2**). In particular, both richness, i.e., observed OTUs and Chao1, and evenness, i.e., Shannon, indexes suggested that not all rhizosphere bacteria have the capacity to thrive on the rhizoplane. This is reminiscent of the selective pressure exerted by roots of land plants on the soil biota (Edwards et al., 2015). We therefore wondered whether this selection pressure was mainly driven by the microhabitat (i.e., rhizoplane or rhizosphere) or by the host species. Strikingly, beta diversity (i.e., between sample diversity) analysis computed with both Bray–Curtis, sensitive to taxa abundances, and weighted UniFrac, sensitive to taxa abundances and relatedness, revealed that impact of microhabitat on the microbiota exceeded the one of the species (**Figure 3**). In particular, rhizosphere and rhizoplane communities retrieved from both species appeared more similar than the community identified in the water surrounding the two plants. Thus, and unlike what has generally been observed for land plants (Bulgarelli et al., 2013), species specificity might play a more limited role in defining the wetland plants microbiota. It is tempting to speculate that, since host species-specific signals, such as root exudates, might get diluted in the wetland environment, microbial capacity to adhere to the root surface might act as a stronger selector for the *Typha* and *Phragmites* microbiota. Importantly, our observations concur with the recent findings of Bowen et al. (2017) who described how phylogenetically related *P. australis* lineages assemble taxonomically congruent rhizosphere microbiota when grown in common garden experiments. To gain further insights into the recruitment cues of the *P. australis* and *T. latifolia* bacterial microbiota we focused on the bacteria significantly enriched in and differentiating rhizoplane communities from the surrounding rhizosphere biota. We identified a subset of bacteria whose enrichment in the rhizoplane appears host species-specific, while another subset of bacteria, with a bias for Actinobacteria, Firmicutes, Planctomycetes, and Proteobacteria appears to be enriched in a microhabitat-responsive (**Figures 4, 5**). Our data suggest that the taxonomic diversity of *Phragmites* and *Typha* root microbiota is taxonomically more complex than previously observed by Li et al. (2013). However, owing to the fact that this latter study and our study focused on geographically separated wetlands (China and Italy, respectively), artificial and natural wetlands, and the different techniques used in the two investigations (low- and high-resolution, respectively), caution should be exerted when comparing these results.

To gain further insights into the spatial organization of the rhizoplane microbiota we subjected root specimens to SEM analysis. We directly observed the rhizoplane microbiota on the root specimens. Two major findings emerged from this analysis. First, we observed microbial matrix-like assemblages on the rhizoplane of both plant species (**Figures 6B,F**) whose adherence to the rhizoplane could be compromised only by two consecutive ultrasound treatments (**Figures 6C,D,G,H**). Second, we observed that the strength of such adhesion to the rhizoplane was influenced by the host species: microbial matrix-like assemblages detected on *P. australis* appeared more stable and resistant to ultrasound treatments than the ones detected on the roots of *T. latifolia* (compare **Figure 6D** with **Figure 6H**). Although the technique used prevented us to generate an accurate enumeration of the bacteria proliferating on the rhizoplane, our results appear in contrast with the findings of Faußer et al. (2012), which showed a greater number of bacterial cells dislocated on root surface of *T. latifolia* respect to *P. australis* plants. These observation suggest that (a) environmental factors contribute to shape the rhizoplane communities organization in wetland plants and (b) that, in these environments, rhizoplane adhesion emerges as a key feature for microbial proliferation. An important prediction of this analysis is the fact that SEM micrographs confirmed that the ultrasound treatment produced, in both tested species, an effective dislodgment of the microbial-like aggregates from the rhizoplane, allowing us to enrich for and characterize members of the rhizoplane microbiota.

The evidence that our specimens were enriched effectively for microbial cells from the root surface motivated us to investigate the culturable portion of communities and their potential ability to form biofilm during three different incubation times. In detail, we isolated 20 morphologically distinct CFUs from *P. australis* and 31 from *T. latifolia* rhizoplane specimens, and only two of them resulted unable to form biofilm in any tested incubation times *in vitro* (**Figure 7**). The majority of them resulted as biofilm formers in at least in one of the tested incubation times. In particular, eight of them maintained a strong ability to form biofilm until later time points. Strikingly, taxonomic identification of 20 individual strains performed using 16S rRNA gene sequencing revealed an overlap between the higher taxonomic ranks (i.e., family and phylum level) of the rhizoplane-enriched microbiota and bacteria identified in the isolation-assay (compare taxonomic assignments of **Figure 5** with the ones of **Figure 7**). Therefore, our data indicate that(a) the biofilm-forming capacity is a distinctive feature of the bacteria isolated from rhizoplane preparations and (b) these bacteria are phylogenetically related to members of the rhizoplane microbiota identified in the Illumina sequencing survey. This becomes evident when looking at members of the Bacillaceae and Oxalobacteraceae families, whose enrichment in the rhizoplane significantly discriminate this compartment from the microbiota surrounding *Phragmites* and *Typha* roots (**Figure 8**). The construction of a more comprehensive bacterial collection for *Phragmites* and *Typha* roots becomes now imperative to test whether properties of specific bacterial isolates are differentially regulated between microhabitat and species. Together, our results suggest that plant-mediated mechanisms and the metabolic potential of individual bacteria thriving in water sculpt the microbiota developing at the *Phragmites* and *Typha* root–soil interface. These findings will set the stage for a rational manipulation of plant–microbiota interactions for phytodepuration improvement.

## Accession Numbers

The Illumina 16S rRNA gene sequences generated in this study are deposited in the European Nucleotide Archive (ENA) under the accession number PRJEB23940. Individual isolates 16S rRNA gene sequences are deposited in GenBank, accession numbers MG687372, MG685831, MG685925, MG693178, MG686091, MG686133, MG686135, MG686234, MG685924, MG693177, MG686083, MG686089, MG687488, MG686090, MG687499, MG686130, MG686134, MG687520, MG687521, and MG687522.

## Author Contributions

LP and GN conceived and designed the experiments. LM performed the scanning electron microscopy analysis of the root samples. LP performed the experiments. LP and DB designed and performed the sequencing data analysis. LP, AB, DB, and GN analyzed the data and wrote the manuscript. All authors discussed the results and commented on the manuscript.

## Conflict of Interest Statement

The authors declare that the research was conducted in the absence of any commercial or financial relationships that could be construed as a potential conflict of interest.
