# Supplementary material for: Unraveling the Composition of the Root-Associated Bacterial Microbiota of Phragmites australis and Typha latifolia
Source: Front Microbiol. 2018 Aug 2;9:1650. doi: 10.3389/fmicb.2018.01650 (PMC6083059; doi:10.3389/fmicb.2018.01650)
Supplement: Supplementary file 2 [file Data_Sheet_2.pdf]

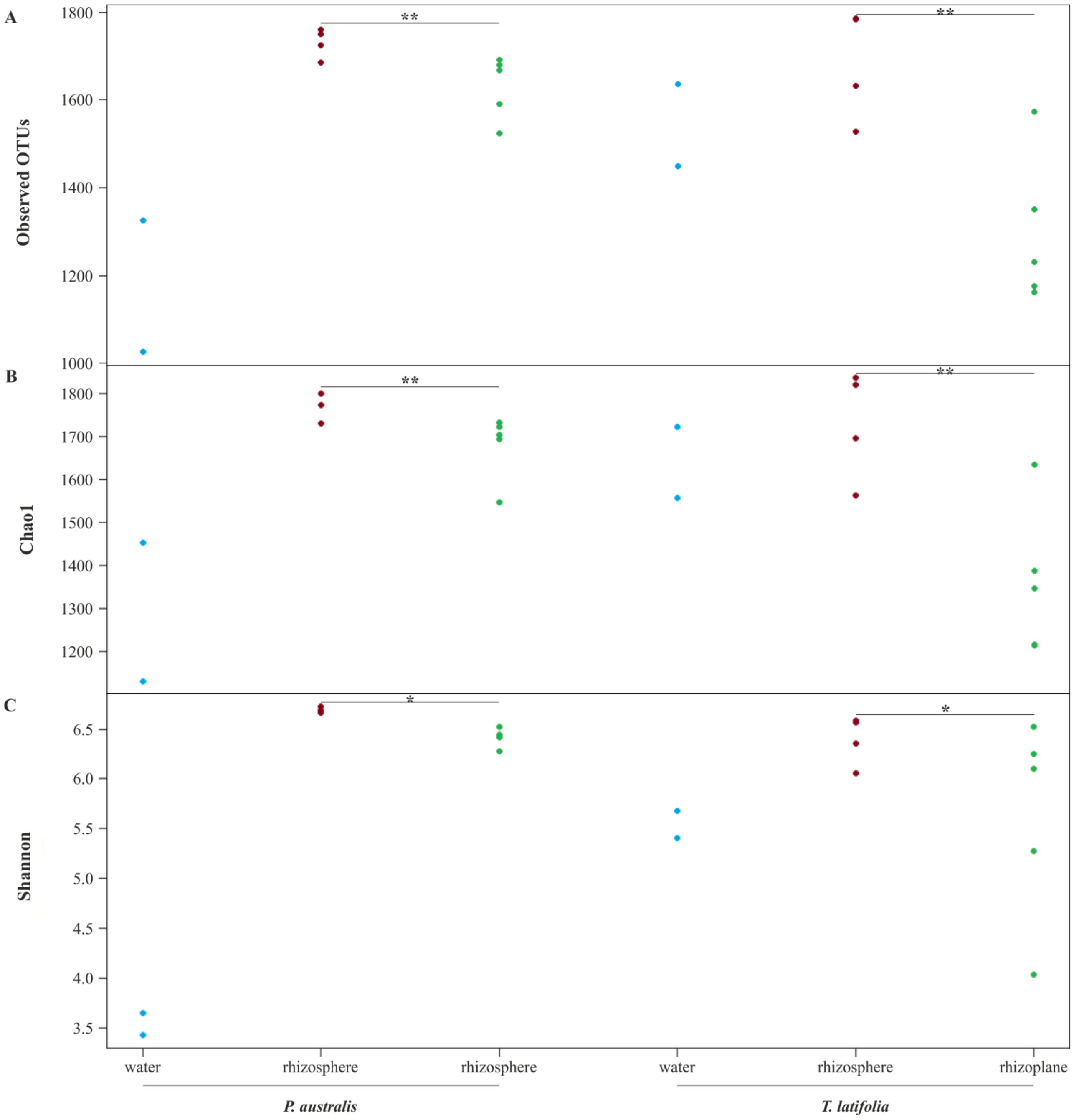

SUPPLEMENTARY FIGURE 2. Alpha diversity calculation for samples set2. OTUs richness of water, rhizosphere and rhizoplane microbiotas of *Phragmites australis* and *Typha latifolia* indicated by number of Observed OTUs **(A)** and by Chao1 index **(B)**. The OTUs eveness of the two plants microbiotas is shown by Shannon index **(C)**. Dots represent single samples. Asterisks denote statistically significant differences between rhizosphere and rhizoplane microhabitats (**$P < 0.01$, * $P < 0.05$).
