# Supplementary material for: Unraveling the Composition of the Root-Associated Bacterial Microbiota of Phragmites australis and Typha latifolia
Source: Front Microbiol. 2018 Aug 2;9:1650. doi: 10.3389/fmicb.2018.01650 (PMC6083059; doi:10.3389/fmicb.2018.01650)
Supplement: Supplementary file 3 [file Data_Sheet_3.pdf]

A

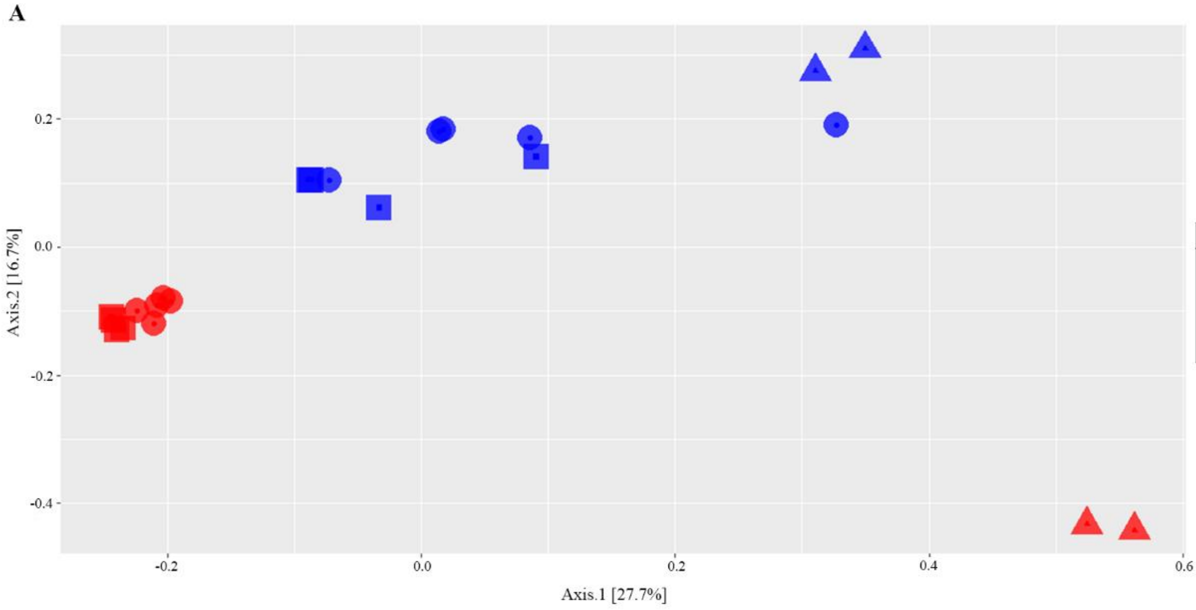

Microhabitat * Species effect		
	R2	P
Microhabitat	33	<0.001
Species	16	<0.001
Microhabitat:Species	16	<0.001

B

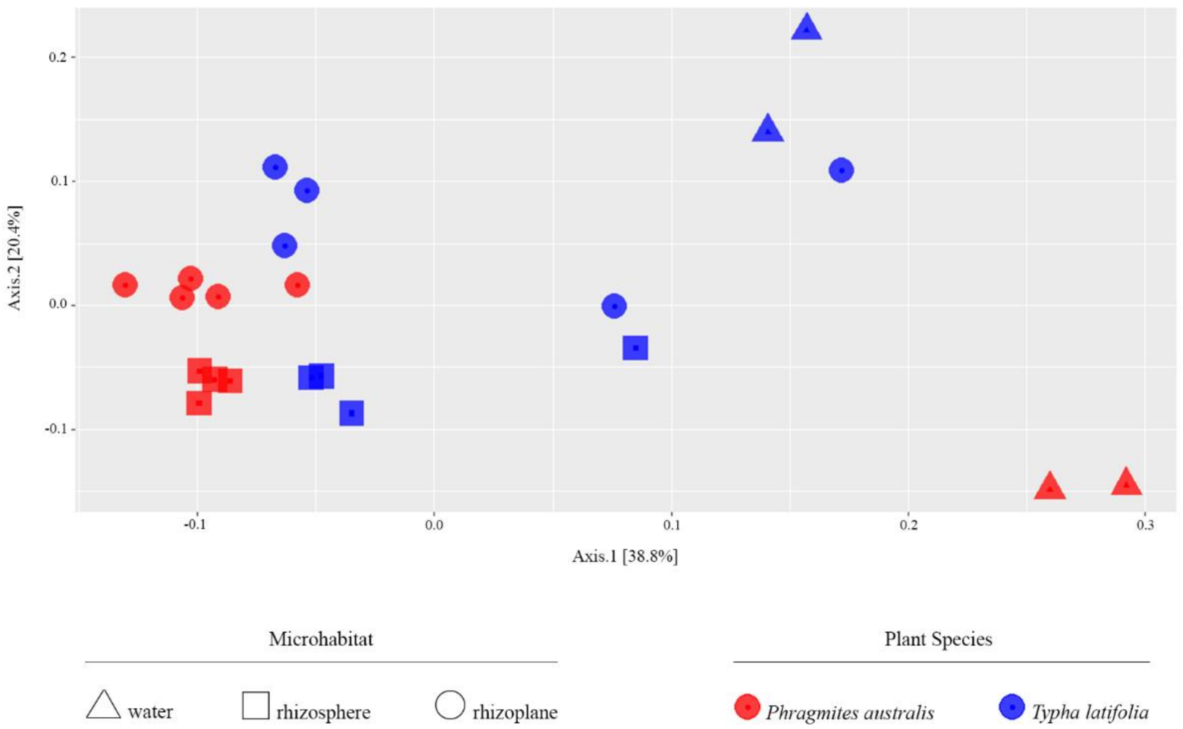

Microhabitat * Species effect		
	R2	P
Microhabitat	39	<0.001
Species	11	<0.001
Microhabitat:Species	18	<0.001

SUPPLEMENTARY FIGURE 3. Beta-diversity calculation for samples set2. On the left, the PCoA plots show the distance between samples calculated on the base of Bray-Curtis index sensitive to the OTUs relative abundance **(A, left)** and on the base of weighted UniFrac index sensitive to both OTU relative abundances and taxonomic affiliation **(B, left)**; the colors of shown symbols depict the plant species and their shapes indicate the considered microhabitats. On the right, the permutational analysis of variances for the indicated sources of variation calculated for the Bray-Curtis **(A, right)** and weighted UniFrac **(B, right)** indexes. The R2 value shows the proportional effect of the indicated factors in the samples distancing and the P-values were calculated for 5,000 permutations.
