# Supplementary material for: Unraveling the Composition of the Root-Associated Bacterial Microbiota of Phragmites australis and Typha latifolia
Source: Front Microbiol. 2018 Aug 2;9:1650. doi: 10.3389/fmicb.2018.01650 (PMC6083059; doi:10.3389/fmicb.2018.01650)
Supplement: Supplementary file 4 [file Image_1.PDF]

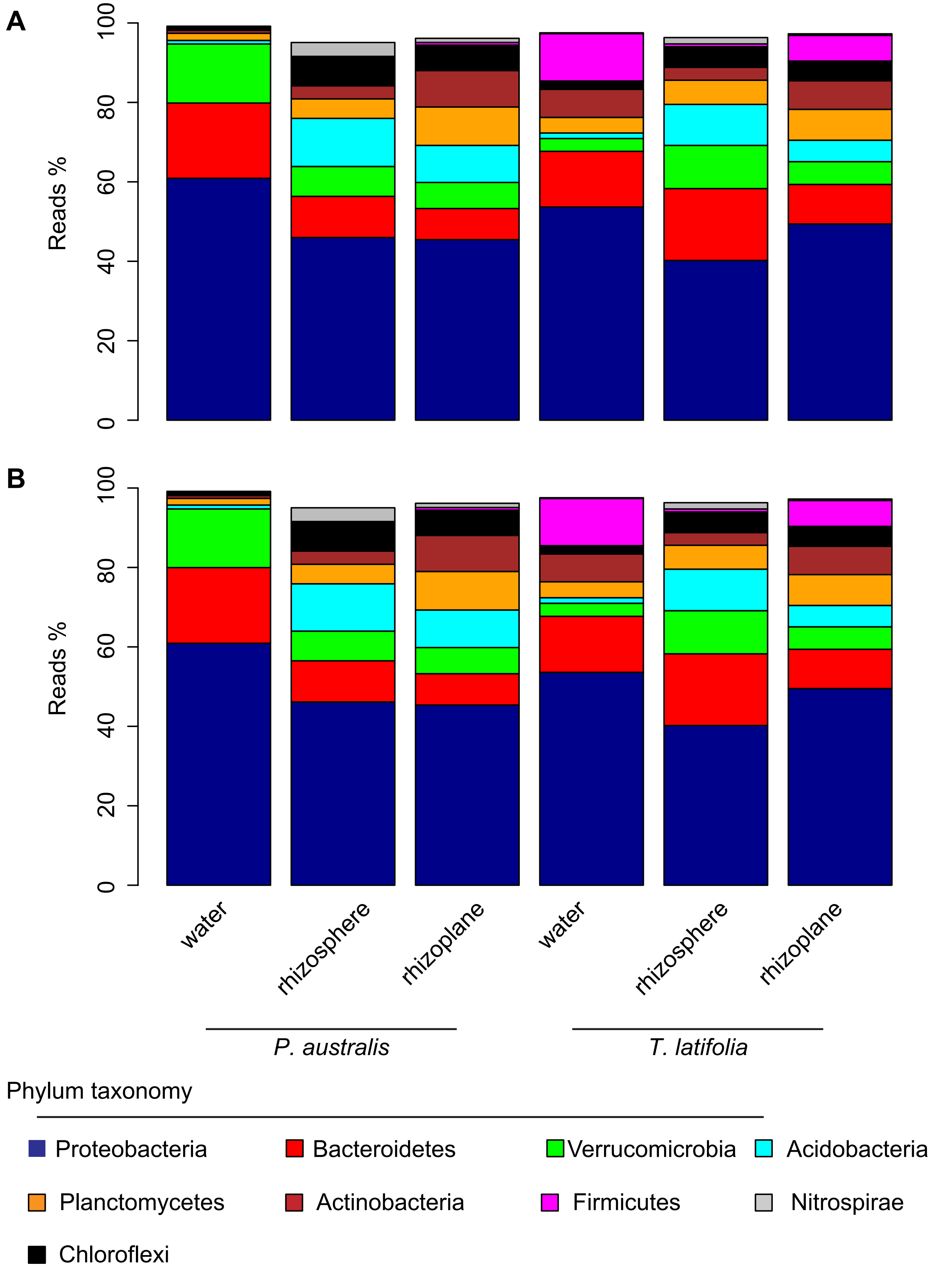

Supplementary Figure 1. Average relative abundace (% of sequencing reads) of the dominant phyla identified in the indicated microhabitats in either *P. australis* or *T. latifolia* in technical replicate set 1 (**A**) or set 2 (**B**), respectively. Only phyla with an average relative abundance of 1% included in the analysis.
