# Supplementary material for: Unraveling the Composition of the Root-Associated Bacterial Microbiota of Phragmites australis and Typha latifolia
Source: Front Microbiol. 2018 Aug 2;9:1650. doi: 10.3389/fmicb.2018.01650 (PMC6083059; doi:10.3389/fmicb.2018.01650)
Supplement: Supplementary file 5 [file Table_1.PDF]

SUPPLEMENTARY TABLE 1. Rhizoplane enriched OTUs. Taxonomical classification of OTUs differentially enriched in the rhizoplane microbiota of each and both plants respect to the rhizosphere.

ENRICHED OTUs IN THE RHIZOPLANE OF						
Phragmites australis						
KINGDOM	PHYLUM	CLASS	ORDER	FAMILY	GENUS	SPECIES
Bacteria	Proteobacteria	Alphaproteobacteria	Rhizobiales	Hyphomicrobiaceae		
Bacteria	Proteobacteria	Alphaproteobacteria	Rhizobiales	Hyphomicrobiaceae	Hyphomicrobium	
Bacteria	Proteobacteria	Alphaproteobacteria	Rhizobiales			
Bacteria	Proteobacteria	Alphaproteobacteria	Rhizobiales			
Bacteria	Proteobacteria	Alphaproteobacteria	Rhizobiales			
Bacteria	Proteobacteria	Alphaproteobacteria	Rhizobiales			
Bacteria	Proteobacteria	Alphaproteobacteria	Rhizobiales			
Bacteria	Proteobacteria	Alphaproteobacteria	Rhizobiales	Hyphomicrobiaceae	Devosia	
Bacteria	Proteobacteria	Alphaproteobacteria	Rhizobiales	Hyphomicrobiaceae	Devosia	
Bacteria	Proteobacteria	Alphaproteobacteria	Rhizobiales	Rhizobiaceae		
Bacteria	Proteobacteria	Alphaproteobacteria	Rhizobiales	Phyllobacteriaceae		
Bacteria	Proteobacteria	Alphaproteobacteria	Rhizobiales	Phyllobacteriaceae		
Bacteria	Proteobacteria	Alphaproteobacteria	Rhizobiales	Methylocystaceae	Methylosinus	
Bacteria	Proteobacteria	Alphaproteobacteria	Rhizobiales	Methylocystaceae	Methylosinus	
Bacteria	Proteobacteria	Alphaproteobacteria	Rhizobiales	Hyphomicrobiaceae	Rhodoplanes	
Bacteria	Proteobacteria	Alphaproteobacteria	Rhizobiales	Bradyrhizobiaceae		
Bacteria	Proteobacteria	Alphaproteobacteria	Rhizobiales			
Bacteria	Proteobacteria	Alphaproteobacteria	Rhizobiales			
Bacteria	Proteobacteria	Alphaproteobacteria	Rhizobiales			
Bacteria	Proteobacteria	Alphaproteobacteria	Rhizobiales			
Bacteria	Proteobacteria	Alphaproteobacteria	Sphingomonadales	Sphingomonadaceae	Novosphingobium	
Bacteria	Proteobacteria	Alphaproteobacteria	Sphingomonadales	Sphingomonadaceae	Novosphingobium	
Bacteria	Proteobacteria	Betaproteobacteria	Burkholderiales	Comamonadaceae	Leptothrix	
Bacteria	Proteobacteria	Betaproteobacteria	Rhodocyclales	Rhodocyclaceae		
Bacteria	Proteobacteria	Betaproteobacteria	Rhodocyclales	Rhodocyclaceae		
Bacteria	Proteobacteria	Betaproteobacteria	Rhodocyclales	Rhodocyclaceae		
Bacteria	Proteobacteria	Betaproteobacteria	Rhodocyclales	Rhodocyclaceae	Sulfuritalea	
Bacteria	Proteobacteria	Betaproteobacteria	Rhodocyclales	Rhodocyclaceae	Sulfuritalea	
Bacteria	Proteobacteria	Betaproteobacteria	Rhodocyclales	Rhodocyclaceae		
Bacteria	Proteobacteria	Betaproteobacteria				
Bacteria	Proteobacteria	Betaproteobacteria				
Bacteria	Proteobacteria	Deltaproteobacteria	Desulfobacterales	Desulfobulbaceae		
Bacteria	Proteobacteria	Deltaproteobacteria	Desulfobacterales	Desulfobulbaceae	Desulfobulbus	
Bacteria	Proteobacteria	Deltaproteobacteria	Desulfuromonadales	Geobacteraceae	Geobacter	
Bacteria	Proteobacteria	Deltaproteobacteria	Myxococcales			
Bacteria	Proteobacteria	Deltaproteobacteria	Myxococcales			
Bacteria	Proteobacteria	Gammaproteobacteria	Legionellales			
Bacteria	Proteobacteria	Gammaproteobacteria	Legionellales	Coxiellaceae	Aquicella	

Bacteria	Proteobacteria	Gammaproteobacteria	Xanthomonadales	Sinobacteraceae	Steroidobacter	
Bacteria	Actinobacteria	Acidimicrobiia	Acidimicrobiales	C111		
Bacteria	Actinobacteria	Acidimicrobiia	Acidimicrobiales	C111		
Bacteria	Actinobacteria	Acidimicrobiia	Acidimicrobiales	C111		
Bacteria	Actinobacteria	Actinobacteria				
Bacteria	Actinobacteria	Actinobacteria	Actinomycetales			
Bacteria	Actinobacteria	Actinobacteria	Actinomycetales			
Bacteria	Actinobacteria	Actinobacteria	Actinomycetales			
Bacteria	Actinobacteria	Actinobacteria	Actinomycetales	Nocardioidaceae	Nocardioides	
Bacteria	Actinobacteria	Actinobacteria	Actinomycetales	Nocardioidaceae		
Bacteria	Actinobacteria	Actinobacteria	Actinomycetales			
Bacteria	Actinobacteria	MB-A2-108	0319-7L14			
Bacteria	Actinobacteria	MB-A2-108	0319-7L14			
Bacteria	Actinobacteria	Thermoleophilia	Gaiellales	Gaiellaceae		
Bacteria	Actinobacteria	Thermoleophilia	Gaiellales	Gaiellaceae		
Bacteria	Actinobacteria	Thermoleophilia	Solirubrobacterales			
Bacteria	Planctomycetes	Planctomycetia	Gemmatales	Gemmataceae		
Bacteria	Planctomycetes	Planctomycetia	Gemmatales	Gemmataceae		
Bacteria	Planctomycetes	Planctomycetia	Gemmatales	Gemmataceae		
Bacteria	Planctomycetes	Planctomycetia	Gemmatales	Gemmataceae	Gemmata	
Bacteria	Planctomycetes	Planctomycetia	Gemmatales	Gemmataceae	Gemmata	
Bacteria	Planctomycetes	Planctomycetia	Pirellulales	Pirellulaceae	Pirellula	
Bacteria	Planctomycetes	Planctomycetia	Pirellulales	Pirellulaceae	Pirellula	
Bacteria	Planctomycetes	Planctomycetia	Pirellulales	Pirellulaceae		
Bacteria	Planctomycetes	Planctomycetia	Pirellulales	Pirellulaceae		
Bacteria	Planctomycetes	Planctomycetia	Pirellulales	Pirellulaceae		
Bacteria	Planctomycetes	Planctomycetia	Pirellulales	Pirellulaceae	A17	
Bacteria	Planctomycetes	Planctomycetia	Pirellulales	Pirellulaceae	A17	
Bacteria	Planctomycetes	Planctomycetia	Planctomycetales	Planctomycetaceae	Planctomyces	
Bacteria	Planctomycetes	Planctomycetia	Planctomycetales	Planctomycetaceae	Planctomyces	
Bacteria	Planctomycetes	Planctomycetia	Planctomycetales	Planctomycetaceae	Planctomyces	
Bacteria	Firmicutes	Bacilli	Bacillales	Bacillaceae	Bacillus	selenatarsenatis
Bacteria	Firmicutes	Bacilli	Bacillales			
Bacteria	Chloroflexi	Ellin6529				
Bacteria	Chloroflexi	Ellin6529				
Bacteria	Chloroflexi	Ellin6529				
Bacteria	Chloroflexi	Ellin6529				
Bacteria	Chloroflexi	Ellin6529				
Bacteria	Chloroflexi	Thermomicrobia	JG30-KF-CM45			
Bacteria	Acidobacteria	Acidobacteria-6	iii1-15			
Bacteria	Acidobacteria	Acidobacteria-6	iii1-15			
Bacteria	Bacteroidetes	[Saprospirae]	[Saprospirales]	Saprospiraceae		
Bacteria	Acidobacteria	[ChlorAcidobacteria]	RB41	Ellin6075		
Bacteria	Verrucomicrobia	Verrucomicrobiae	Verrucomicrobiales	Verrucomicrobiaceae	Luteolibacter	
Bacteria	Verrucomicrobia	Verrucomicrobiae	Verrucomicrobiales	Verrucomicrobiaceae		
Bacteria	Verrucomicrobia	Verrucomicrobiae	Verrucomicrobiales	Verrucomicrobiaceae		

Bacteria	Verrucomicrobia	[Spartobacteria]	[Chthoniobacterales]	[Chthoniobacteraceae]	Candidatus Xiphinematobacter	

Typha latifolia

KINGDOM	PHYLUM	CLASS	ORDER	FAMILY	GENUS	SPECIES
Bacteria	Proteobacteria	Alphaproteobacteria	Rhizobiales	Hyphomicrobiaceae	Hyphomicrobium	
Bacteria	Proteobacteria	Alphaproteobacteria	Rhizobiales			
Bacteria	Proteobacteria	Alphaproteobacteria	Rhizobiales			
Bacteria	Proteobacteria	Alphaproteobacteria	Rhizobiales			
Bacteria	Proteobacteria	Alphaproteobacteria	Rhizobiales			
Bacteria	Proteobacteria	Alphaproteobacteria	Rhizobiales	Rhizobiaceae		
Bacteria	Proteobacteria	Alphaproteobacteria	Rhizobiales	Phyllobacteriaceae		
Bacteria	Proteobacteria	Alphaproteobacteria	Rhizobiales	Methylocystaceae	Pleomorphomonas	
Bacteria	Proteobacteria	Alphaproteobacteria	Rhizobiales			
Bacteria	Proteobacteria	Alphaproteobacteria	Rhizobiales	Methylocystaceae	Methylosinus	
Bacteria	Proteobacteria	Alphaproteobacteria	Rhizobiales	Methylocystaceae	Methylosinus	
Bacteria	Proteobacteria	Alphaproteobacteria	Rhizobiales			
Bacteria	Proteobacteria	Alphaproteobacteria	Rhizobiales			
Bacteria	Proteobacteria	Alphaproteobacteria	Rhizobiales			
Bacteria	Proteobacteria	Alphaproteobacteria	Rhizobiales			
Bacteria	Proteobacteria	Alphaproteobacteria	Rhodobacterales	Rhodobacteraceae		
Bacteria	Proteobacteria	Alphaproteobacteria	Rhodobacterales	Rhodobacteraceae		
Bacteria	Proteobacteria	Alphaproteobacteria	Rhodobacterales	Rhodobacteraceae	Rhodobacter	
Bacteria	Proteobacteria	Alphaproteobacteria	Rhodobacterales	Rhodobacteraceae	Rhodobacter	
Bacteria	Proteobacteria	Alphaproteobacteria	Sphingomonadales	Sphingomonadaceae	Novosphingobium	
Bacteria	Proteobacteria	Betaproteobacteria	Methylophilales	Methylophilaceae		
Bacteria	Proteobacteria	Betaproteobacteria	SC-I-84			
Bacteria	Proteobacteria	Betaproteobacteria	SC-I-84			
Bacteria	Proteobacteria	Gammaproteobacteria	Pseudomonadales	Pseudomonadaceae	Pseudomonas	viridiflava
Bacteria	Proteobacteria	Gammaproteobacteria	Pseudomonadales	Moraxellaceae	Acinetobacter	johnsonii
Bacteria	Proteobacteria	Gammaproteobacteria	Pseudomonadales	Moraxellaceae	Acinetobacter	rhizosphaerae
Bacteria	Proteobacteria	Gammaproteobacteria	Pseudomonadales	Moraxellaceae	Acinetobacter	
Bacteria	Actinobacteria	Acidimicrobiia	Acidimicrobiales	C111		
Bacteria	Actinobacteria	Acidimicrobiia	Acidimicrobiales	C111		
Bacteria	Actinobacteria	Acidimicrobiia	Acidimicrobiales	C111		
Bacteria	Actinobacteria	Acidimicrobiia	Acidimicrobiales	C111		
Bacteria	Actinobacteria	Acidimicrobiia	Acidimicrobiales	C111		
Bacteria	Actinobacteria	Acidimicrobiia	Acidimicrobiales	Microthrixaceae		
Bacteria	Actinobacteria	Actinobacteria	Actinomycetales	Streptomycetaceae		
Bacteria	Actinobacteria	Actinobacteria	Actinomycetales			
Bacteria	Actinobacteria	Actinobacteria	Actinomycetales	Mycobacteriaceae	Mycobacterium	
Bacteria	Actinobacteria	Actinobacteria	Actinomycetales	Mycobacteriaceae	Mycobacterium	
Bacteria	Actinobacteria	Actinobacteria	Actinomycetales	Mycobacteriaceae	Mycobacterium	
Bacteria	Actinobacteria	Actinobacteria	Actinomycetales	Nocardioidaceae		
Bacteria	Actinobacteria	Actinobacteria	Actinomycetales			
Bacteria	Actinobacteria	Thermoleophilia	Gaiellales	Gaiellaceae		
Bacteria	Actinobacteria	Thermoleophilia	Solirubrobacterales			

Bacteria	Planctomycetes	Planctomycetia	Gemmatales	Gemmataceae		
Bacteria	Planctomycetes	Planctomycetia	Gemmatales	Gemmataceae	Gemmata	
Bacteria	Planctomycetes	Planctomycetia	Gemmatales	Gemmataceae	Gemmata	
Bacteria	Planctomycetes	Planctomycetia	Gemmatales	Gemmataceae	Gemmata	
Bacteria	Planctomycetes	Planctomycetia	Gemmatales	Gemmataceae	Gemmata	
Bacteria	Planctomycetes	Planctomycetia	Gemmatales	Isosphaeraceae		
Bacteria	Planctomycetes	Planctomycetia	Pirellulales	Pirellulaceae		
Bacteria	Planctomycetes	Planctomycetia	Pirellulales	Pirellulaceae	A17	
Bacteria	Firmicutes	Bacilli	Bacillales	Alicyclobacillaceae	Alicyclobacillus	
Bacteria	Firmicutes	Bacilli	Bacillales	Bacillaceae	Bacillus	selenatarsenatis
Bacteria	Firmicutes	Bacilli	Bacillales			
Bacteria	Firmicutes	Bacilli	Bacillales			
Bacteria	Firmicutes	Bacilli	Bacillales			
Bacteria	Firmicutes	Bacilli	Bacillales	[Exiguobacteraceae]	Exiguobacterium	
Bacteria	Firmicutes	Bacilli	Bacillales	[Exiguobacteraceae]	Exiguobacterium	
Bacteria	Firmicutes	Bacilli	Bacillales	Planococcaceae		
Bacteria	Firmicutes	Clostridia	Clostridiales	Peptostreptococcaceae		
Bacteria	Chloroflexi	Anaerolineae	Caldilineales	Caldilineaceae	Caldilinea	
Bacteria	Chloroflexi	Chloroflexi	[Roseiflexales]	[Kouleothrixaceae]		
Bacteria	Chloroflexi	Chloroflexi	[Roseiflexales]	[Kouleothrixaceae]		
Bacteria	Acidobacteria	Acidobacteria-6	iii1-15			
Bacteria	Acidobacteria	Acidobacteria-6	iii1-15			
Bacteria	Acidobacteria	[ChlorAcidobacteria]	DS-100			
Bacteria	Acidobacteria	Sva0725	Sva0725			
Bacteria	Acidobacteria	Sva0725	Sva0725			
Phragmites australis* and *Typha latifolia						
KINGDOM	PHYLUM	CLASS	ORDER	FAMILY	GENUS	SPECIES
Bacteria	Proteobacteria	Alphaproteobacteria	Rhizobiales			
Bacteria	Proteobacteria	Alphaproteobacteria	Rhizobiales			
Bacteria	Proteobacteria	Alphaproteobacteria	Rhizobiales			
Bacteria	Proteobacteria	Alphaproteobacteria	Rhizobiales	Rhizobiaceae		
Bacteria	Proteobacteria	Alphaproteobacteria	Rhizobiales	Phyllobacteriaceae		
Bacteria	Proteobacteria	Alphaproteobacteria	Rhizobiales	Methylocystaceae	Methylosinus	
Bacteria	Proteobacteria	Alphaproteobacteria	Rhizobiales	Methylocystaceae	Methylosinus	
Bacteria	Proteobacteria	Alphaproteobacteria	Rhizobiales			
Bacteria	Actinobacteria	Acidimicrobiia	Acidimicrobiales	C111		
Bacteria	Actinobacteria	Acidimicrobiia	Acidimicrobiales	C111		
Bacteria	Actinobacteria	Acidimicrobiia	Acidimicrobiales	C111		
Bacteria	Actinobacteria	Actinobacteria	Actinomycetales			
Bacteria	Actinobacteria	Actinobacteria	Actinomycetales	Nocardioidaceae		
Bacteria	Actinobacteria	Actinobacteria	Actinomycetales			
Bacteria	Actinobacteria	Thermoleophilia	Gaiellales	Gaiellaceae		
Bacteria	Actinobacteria	Thermoleophilia	Solirubrobacterales			
Bacteria	Planctomycetes	Planctomycetia	Gemmatales	Gemmataceae		
Bacteria	Planctomycetes	Planctomycetia	Gemmatales	Gemmataceae	Gemmata	

Bacteria	Planctomycetes	Planctomycetia	Pirellulales	Pirellulaceae		
Bacteria	Planctomycetes	Planctomycetia	Pirellulales	Pirellulaceae	A17	
Bacteria	Firmicutes	Bacilli	Bacillales	Bacillaceae	Bacillus	selenatarsenatis
Bacteria	Firmicutes	Bacilli	Bacillales			
