# Supplementary material for: Unraveling the Composition of the Root-Associated Bacterial Microbiota of Phragmites australis and Typha latifolia
Source: Front Microbiol. 2018 Aug 2;9:1650. doi: 10.3389/fmicb.2018.01650 (PMC6083059; doi:10.3389/fmicb.2018.01650)
Supplement: Supplementary file 6 [file Table_2.PDF]

| ISOLATE | GREENGENES OTUs | TAXONOMICAL CLASSIFICATION | | | | | | |
|---|---|---|---|---|---|---|---|---|
| | | Kingdom | Phylum | Class | Order | Family | Genus | Species |
| P3 | 580010 | Bacteria | Firmicutes | Bacilli | Bacillales | Bacillaceae | Bacillus | |
| P6; P9; P15 | 357169 | Bacteria | Firmicutes | Bacilli | Bacillales | Bacillaceae | Bacillus | |
| T12; T4 | 2034540 | Bacteria | Firmicutes | Bacilli | Bacillales | Bacillaceae | | |
| T13 | 3599421 | Bacteria | Firmicutes | Bacilli | Bacillales | Bacillaceae | | |
| P14 | 412145 | Bacteria | Firmicutes | Bacilli | Bacillales | Staphylococcaceae | Staphylococcus | |
| T8 | 4458776 | Bacteria | Actinobacteria | Actinobacteria | Actinomycetales | Microbacteriaceae | Microbacterium | chocolatum |
| T16 | 535932 | Bacteria | Actinobacteria | Actinobacteria | Actinomycetales | Microbacteriaceae | Microbacterium | chocolatum |
| T24 | 3017908 | Bacteria | Actinobacteria | Actinobacteria | Actinomycetales | Nocardiaceae | Rhodococcus | |
| T30 | 1129906 | Bacteria | Bacteroidetes | Flavobacteriia | Flavobacteriales | [Weeksellaceae] | Wautersiella | |
| P4 | 341259 | Bacteria | Proteobacteria | Betaproteobacteria | Burkholderiales | Comamonadaceae | | |
| T1 | 681779 | Bacteria | Proteobacteria | Gammaproteobacteria | Enterobacteriales | Enterobacteriaceae | | |
| P13 | 1108343 | Bacteria | Firmicutes | Bacilli | Bacillales | [Exiguobacteraceae] | Exiguobacterium | |
| T6 | 536390 | Bacteria | Proteobacteria | Gammaproteobacteria | Pseudomonadales | Pseudomonadaceae | Pseudomonas | |
| T10 | 750541 | Bacteria | Proteobacteria | Gammaproteobacteria | Xanthomonadales | Xanthomonadaceae | | |
| T20 | 237591 | Bacteria | Proteobacteria | Betaproteobacteria | Burkholderiales | Oxalobacteraceae | Janthinobacterium | lividum |
| T21 | 920852 | Bacteria | Actinobacteria | Actinobacteria | Actinomycetales | Streptomycetaceae | Streptomyces | mirabilis |
| T23 | 582591 | Bacteria | Actinobacteria | Actinobacteria | Actinomycetales | Streptomycetaceae | Streptomyces | |

**SUPPLEMENTARY TABLE 2. Taxonomy of rhizoplane isolates.** Taxonomical classification of rhizoplane isolates with the correspondent OTU code from Greengenes database.
